# Supplementary figures and images for: Identifying Network Perturbation in Cancer
Source: PLoS Comput Biol. 2016 May 4;12(5):e1004888. doi: 10.1371/journal.pcbi.1004888 (PMC4856318; doi:10.1371/journal.pcbi.1004888)

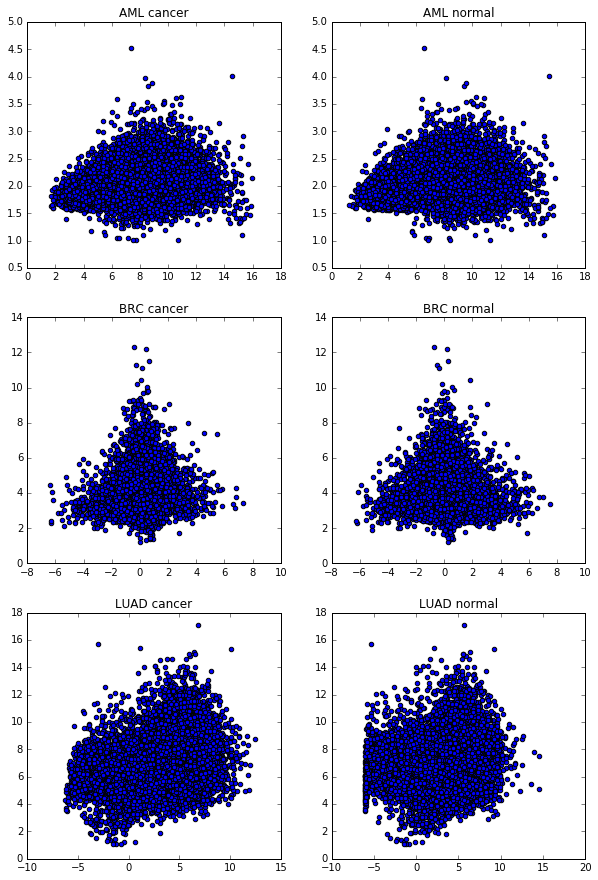

Supplement: S1 Fig — We show that genes with very low mean expression levels do not tend to have high enough DISCERN score to be considered in our analysis. The Pearson’s correlation between the mean expression before standardization and the DISCERN score ranges from 0.08 and 0.43. Positive correlation is induced because genes with very low mean expression tend to have lower DISCERN scores, indicating that there is probably not an issue in terms of overly selecting genes whose expression are essentially noise. In LUAD, the voom normalization method removes many genes with low expression. We can see that many dots were removed between -5 and -10 for LUAD (bottom). (TIFF) [file pcbi.1004888.s002.tiff]
